# Immunization supply chains: Why they matter and how they are changing☆

**DOI:** 10.1016/j.vaccine.2017.02.062

**Published:** 2017-04-19

**Authors:** Raja Rao, Benjamin Schreiber, Bruce Y. Lee

**Affiliations:** Bill & Melinda Gates Foundation, Seattle, WA, USA; United Nations Children’s Fund, New York, NY, USA; Department of International Health, Johns Hopkins Bloomberg School of Public Health, Baltimore, MD, USA; Johns Hopkins Carey Business School, Baltimore, MD, USA

Considerable investments have been made over the past two decades to ensure that children and adults throughout the world have equitable access to safe and effective vaccines. New vaccines, formulations, delivery devices, and cold chain technologies have made it possible for developing countries to protect millions of people from some of the world’s deadliest infectious diseases. Novel financing mechanisms have made new vaccines more affordable, and global attention toward immunization is at an all-time high. However, comparatively few investments have been made to ensure that immunization products are safely and reliably managed and delivered from the point of arrival in a country to the many places where immunization services are provided.

The daily struggle to deliver vaccines to communities is a concern and source of frustration for many people, including the readers of *Vaccine*, from bench researchers to vaccine manufacturers to epidemiologists, donors, and public health practitioners. Why? Because poorly designed and executed delivery systems are delaying and limiting the impact that vaccines have on population health. Unresolved supply chain issues are stalling new vaccine introductions [1], contributing to prolonged vaccine stockouts due to forecasting errors [5], wasting vaccines by accidentally exposing them to freezing or too warm temperatures [6], and constraining coverage by vaccines not being available when and where they are needed [2,7]. After all of the money, breakthrough science, and technological ingenuity that has gone into vaccine research and development, one might wonder: what is so difficult about the logistics of vaccine delivery? Yes, roads are bad and health workers are overworked, electricity comes and goes, vehicles and fridges break down, etc. But these problems should be solvable.

As Lloyd and Cheyne [8] explain in their article on the history of the vaccine supply chain, supply chains were designed for a different era in immunization. They have evolved slowly, with minimal investment. Knowing that by 2020 vaccines will comprise 40% of an average immunization budget [3], tolerance for inefficient and wasteful supply chains is quickly waning. Countries are aware that fundamental changes must be made to the way supply chains are designed, equipped, staffed, and managed and how information and funding flow from one end to the other. The question is, ‘‘how?” There is a dearth of evidence that links supply chain improvements to immunization coverage and health impact. In addition, few countries are aware of the successful impact that certain proposed solutions have had, and which countries are adopting them in an effort to meet coverage and equity goals.

By focusing entirely on the vaccine supply chain, this special issue of *Vaccine* compiles the latest evidence on the opportunities and challenges facing immunization supply chains, including the costs and benefits of various solutions that have been proposed and piloted in developing-county contexts. Not only does it serve as a ready reference, it underscores the important role that immunization supply chains play in achieving higher and more equitable immunization coverage.

Members of Gavi, the Vaccine Alliance, have articulated the complex, interdependencies inherent to supply chain systems and identified five fundamental areas that must be improved as we develop the next-generation of immunization supply chains: leadership, oversight, design, data, and cold chain equipment. Interestingly, while these five fundamental areas all focus on country-level processes, each area can be impacted by decisions made in the earliest discovery phases of vaccine development, all the way to the implementation phases of vaccine deployment.

## Leadership

While a majority of countries have supply chain managers in place, few such managers have been professionally trained or educated in supply chain management, nor do they always have the authority, accountability, or fiduciary control to improve supply chain performance [9]. Ministers of Health and immunization program managers may be interested in the growing body of education and training programs now available for supply chain and logistics professionals [10] and the ways in which trained personnel have been used to make a difference in supply chain performance (Huong et al.), [11–14].

## Continuous improvement

The Effective Vaccine Management (EVM) process benchmarks supply chain performance against best practices in nine areas of vaccine management at each level of the health system. To date, only a handful of countries have achieved average scores above the desired threshold of 80% [15]. National program managers and country-based agencies may be interested in the oversight mechanisms that other countries have established to monitor performance and recommend policies changes to improve supply chain performance [16].

## System design

Most vaccine supply chains were designed to follow the administrative hierarchy of the health system, relying on overburdened health and medical staff to perform logistics functions. Recognizing that different delivery, storage, and staffing configurations might yield better results, countries like Benin, Nigeria, India, and Mozambique have fundamentally redesigned their supply chains in pilot regions, introducing new staff positions, new cold chain equipment, and new logistics data systems to improve performance. Decision-makers in other lower income countries may be inspired to learn that changes made in these regions have significantly reduced facility-level stockouts, improved staff satisfaction, and increased rates of vaccine availability, translating to improved coverage, and in some cases reduced costs (Huong et al.), [11–14,17].

## Logistics data

Incomplete and inaccurate data collection systems have long stymied immunization programs, making it nearly impossible to use data to forecast vaccine requirements and deliver vaccines when and where they are needed. According to research by Lydon et al. [5], vaccine stockouts lasting one month or longer occur in one of every three countries globally, and about 89% of these national stockouts compromise vaccine availability at the service delivery level. Countries considering electronic data systems may be interested in learning about new electronic data systems that have been piloted and scaled in developing countries, including a new system in Uttar Pradesh, India, which was quickly adopted and led to reduced stockouts and increased vaccine availability after 13 months of consistent use [14]. Procurement staff may wish to learn how data on vaccine wastage and session size is being used to determine optimal dose-per-vial in specific country situations [18].

### COLD CHAIN

Accidental freezing still occurs in 33% of storage facilities in wealthy countries and 37% of facilities in lower income countries [6], and cold chain equipment is failing and underperforming in 20 and 50% (respectively) of 55 Gavi-eligible countries [19]. Solutions to these problems are being developed at many different levels and with different time horizons in mind [20]. Vaccine manufacturers may be interested in formulations that can withstand freezing [4], and program managers my want to know how to use vaccines in a controlled temperature chain [21]. National immunization staff and procurement officers may consider the value of cold chain equipment that is designed to avoid freezing and to operate successfully in areas without reliable access to electricity [20] (Franzel et al.). Program managers may find it surprising to know that their investigations of equipment failures can yield valuable and actionable information for technicians, procurement officers, equipment manufacturers, and international partners [22].

With this special issue, we hope that the full array of stakeholders involved in vaccine discovery, development, and deployment will find reasons to continue innovating and adopting practical solutions to downstream challenges of vaccine delivery. Without a proactive and collective response from immunization stakeholders, no product being developed now will have the impact that it should. The challenges of vaccine distribution, storage, and delivery described in this special issue are complex, but solvable. Many solutions have been piloted and some have been scaled up, and we expect that these innovations will drive further investment in the long-neglected and underfunded area of immunization supply chains.
